# In vitro antiviral effect of ethanolic extracts from *Azadirachta indica* and *Melia azedarach* against goat lentivirus in colostrum and milk

**DOI:** 10.1038/s41598-023-31455-5

**Published:** 2023-03-22

**Authors:** Ana Lidia Madeira de Sousa, Raymundo Rizaldo Pinheiro, Juscilania Furtado Araujo, Renato Mesquita Peixoto, Dalva Alana Aragao de Azevedo, Ana Milena Cesar Lima, Kirley Marques Canuto, Paulo Riceli Vasconcelos Ribeiro, Ana Sheila de Queiroz Souza, Samara Cristina Rocha Souza, Sara Lucena de Amorim, Gabriel Paula Amaral, Viviane de Souza, Selene Maia de Morais, Alice Andrioli, Maria Fatima da Silva Teixeira

**Affiliations:** 1 Laboratory of Virology (LABOVIR), State University of Ceará (UECE), Fortaleza, CE Brazil; 2 Faculdade Educar da Ibiapaba, Ípu, CE Brazil; 3 Laboratory of Virology, Embrapa Goats and Sheep, Sobral, CE Brazil; 4 University Center INTA (UNINTA), Tianguá, CE Brazil; 5 Vale do Salgado University Center (UNIVS), Icó, CE Brazil; 6 Terra Nordeste College (FATENE), Caucaia, CE Brazil; 7 School of Permanent Training for Teaching and Educational Management, Sobral, CE Brazil; 8 Scholarship for Regional Scientific Development of the National Council for Scientific and Technological Development (DCR-CNPq/FUNCAP), Level C, Embrapa Goats and Sheep, Sobral, CE Brazil; 9 Multiuser Laboratory of Natural Products Chemistry, Embrapa Tropical Agroindustry, Fortaleza, CE Brazil; 10 Department of Veterinary Medicine, Federal University of Rondônia, Rolim de Moura, RO Brazil; 11 State University of Acaraú Valley, Sobral, CE Brazil; 12 Laboratory of Microbiology, Embrapa Goats and Sheep, Sobral, CE Brazil; 13 Laboratory of Chemistry and Natural Products (LQPN), Ceará State University, Fortaleza, CE Brazil

**Keywords:** Virology, Antivirals, Viral transmission

## Abstract

This study aimed to evaluate, in vitro, the use of leaf extracts of *Azadirachta indica* (*A. indica*) and *Melia azedarach* (*M. azedarach*) as antivirals against caprine lentivirus (CLV) in colostrum and milk of goat nannies. These were collected from eight individuals and infected with the standard strain of CLV. Samples were then subdivided into aliquots and treated with 150 µg/mL of crude extract, and with ethyl acetate and methanol fractions for 30, 60, and 90 min. Next, somatic cells from colostrum and milk were co-cultured with cells from the ovine third eyelid. After this step, viral titers of the supernatants collected from treatments with greater efficacy in co-culture were assessed. The organic ethyl acetate fractions of both plants at 90 min possibly inhibited the viral activity of CLV by up to a thousandfold in colostrum. In milk, this inhibition was up to 800 times for the respective Meliaceae. In conclusion, the ethanolic fraction of ethyl acetate from both plants demonstrated efficacy against CLV in samples from colostrum and milk when subjected to treatment, which was more effective in colostrum.

## Introduction

Small Ruminant Lentiviruses (SRLV) constitute a wide phylogenetic group of retroviruses^1^. These are commonly divided into Caprine Lentivirus (CLV), which includes strains that cause Caprine Arthritis Encephalitis (CAE)^2^ in goats, and Ovine Lentivirus (OLV), which includes those that cause Maedi-Visna (MV) disease in sheep^3,4^. However, these viral agents have high mutagenic potential and frequently cross the interspecific barrier between ruminants^5,6^. In addition, these infections are contagious and incurable diseases that cause significant economic losses in goat and sheep production^3,7,8^. Currently, there are no effective treatments or vaccines available^9,10^, and the search for novel prevention strategies is necessary. The CAE virus can be transmitted in several ways. Among these, the lactogenic pathway is an important mechanism by which this disease may pass between animals, and may occur through the ingestion of infected colostrum and milk, either by free viral particles or by viruses contained in monocytes/macrophages^11,12^. Hence, blocking this form of transmission is important because of the large dissemination of the virus in flocks^13,14^. Notable among these techniques is the use of artificial colostrum, which is composed of 700 mL of healthy bovine milk, 300 mL of blood serum from negative goats or sheep, and a hen’s egg^15^. It is also recommended to use (healthy) cow colostrum to feed goats and sheep^15,16^. The pasteurization of milk can also be mentioned, as it maintains the organoleptic and nutritional characteristics, while ensuring the destruction of pathogenic microorganisms, making it a healthy food^17^. Another widely used method is the thermization procedure, which can be performed on colostrum, transitional milk, and regular milk, in which these are heated in a water bath at 56 °C for one hour and then stored at – 15 °C^16,18^. However, this method requires equipment and trained staff, making it laborious to execute^18^. Therefore, it is important to research new practical alternatives that are low cost for producers and are effective in preventing dissemination of the disease via the lactogenic pathway in flocks.

In this context, validation of antiviral agents for the treatment of curable, incurable, chronic, and acute viral infections is in constant evolution^19–23^. Among medicines, plants of the Meliaceae family, such as *A. indica* (neem) and *M. azedarach* (chinaberry tree), have demonstrated several applications in human and veterinary medicine, including as insecticides, bactericides and antivirals^22–24^. Leaf extracts of *A. indica* were tested against the human immunodeficiency virus (HIV) in the form of vaginal tablets, and promising results for the control and dissemination of this pathogen in India were found^25^. In addition, neem demonstrated effects on the dengue virus, inhibiting protease activity by means of bioflavonoids, which could contribute to the development of an effective drug against viral infection^24^.

*Melia azedarach* inhibits the multiplication of herpes simplex virus type 2 (HSV-2) in epithelial cells and increases the cytokine production in macrophages, which are important traits for viral elimination^26^*.* Furthermore, it showed potent antiviral activity against several strains of influenza virus (H_5_N_1_, H_1_N_1_, H_3_N_2_, H_7_N_9_ and H_9_N_2_) in in vitro and in vivo studies^23^. Therefore, this study aimed to evaluate in vitro the use of ethanolic leaf extracts of *A. indica* and *M. azedarach* as antiviral agents against CLV in goat colostrum and milk.

## Results

### Toxicological analysis by mean lethal concentration (LC_50_) in ***Artemia salina*** Leach

After carrying out toxicity tests with *Artemia salina* Leach (*A. salina*), the LC50 of all treatments was established [Control, CEE-AI (crude ethanolic extract—*Azadirachta indica*), EAF-AI (ethyl acetate fraction—*Azadirachta indica*), MF-AI (fraction methanol—*Azadirachta indica*), CEE-MA (crude ethanolic extract—*Melia azedarach*), EAF-MA (ethyl acetate fraction—*Melia azedarach*), MF-MA (fraction methanol—*Melia azedarach*)], and their respective estimated concentrations (µg/mL), in the period of 24 h, 48 h and 72 h (Table 1). The LC_50_ values obtained for *A. indica* in CEE-AI and its EAF-AI and MF-AI fractions were 210.614, 160.562, and 155.715 µg/mL, respectively, in the 24 h period. Taking into account this same period, the estimated values in the LC50 of *M. azedarach* in the CEE-MA, EAF-MA, and MF-MA fractions were 109.134 (with maximum use limits of 207.826 µg/mL), 179.099 (with maximum use limits of 334.472 µg/mL), and 147.350 µg/mL (with maximum use limits of 268.793 µg/mL), respectively.

**Table 1:** Estimating the mean lethal concentration (LC_50_) of crude extracts of *Azadirachta indica* and *Melia azedarach* their respective fractions of ethyl acetate and methanol in *Artemia salina* Leach with 95% confidence limits for concentration (µg/mL).

Time	No	Group	95% confidence limits for concentration (µg/mL)			95% confidence limits for log_10_ (concentration (µg/mL))		
			Estimates	Inferior limit	Upper limit	Estimates	Inferior limit	Upper limit
24 h	1	Control	26.095	6.334	87.404	1.417	0.802	1.942
	2	CEE-AI	210.614	101.103	393.452	2.323	2.005	2.595
	3	EAF-AI	160.562	80.729	292.210	2.206	1.907	2.466
	4	MF-AI	155.715	69.440	296.115	2.192	1.842	2.471
	5	CEE-MA	109.134	46.774	207.826	2.038	1.670	2.318
	6	EAF-MA	179.099	87.020	334.472	2.253	1.940	2.524
	7	MF-MA	147.350	71.409	268.793	2.168	1.854	2.429
48 h	8	Control	1.771	0.002	19.937	0.248	− 2.683	1.300
	9	CEE-AI	2.324	0.001	20.157	0.366	− 2.930	1.304
	10	EAF-AI	2.163	0.002	16.185	0.335	− 2.739	1.209
	11	MF-AI	1.690	0	17.387	0.228	− 3.352	1.240
	12	CEE-MA	1.169	0	12.709	0.068	− 3.618	1.104
	13	EAF-MA	0.37	0	5.894	− 0.432	− 4.689	0.77
	14	MF-MA	0.313	0	5.250	− 0.504	− 4.875	0.72
72 h	15	Control	0.313	0	5.250	− 0.504	− 4.875	0.72
	16	CEE-AI	0	–	–	− 7.773	–	–
	17	EAF-AI	0	–	–	− 7.716	–	–
	18	MF-AI	0	–	–	− 7.754	–	–
	19	CEE-MA	0	–	–	− 10.446	–	–
	20	EAF-MA	0	–	–	− 10.215	–	–
	21	MF-MA	0	–	–	− 9.839	–	–

CEE-AI: crude ethanolic extract—*Azadirachta indica*; EAF-AI: ethyl acetate fraction—*Azadirachta indica*; MF-AI: fraction methanol—*Azadirachta indica*; CEE-MA: crude ethanolic extract—*Melia azedarach*; EAF-MA: ethyl acetate fraction—*Melia azedarach*; MF-MA: fraction methanol—*Melia azedarach.*

After 24 h, an increase in the death of crustaceans was evident with low dosages of the extracts of both Meliaceae. For the control treatment, the LC_50_ related to the concentration of NaCl was estimated to be 26.095 g/L; it should be noted that only the concentration of NaCl was estimated because it is the only component present in the treatment.

**Figure 1:**
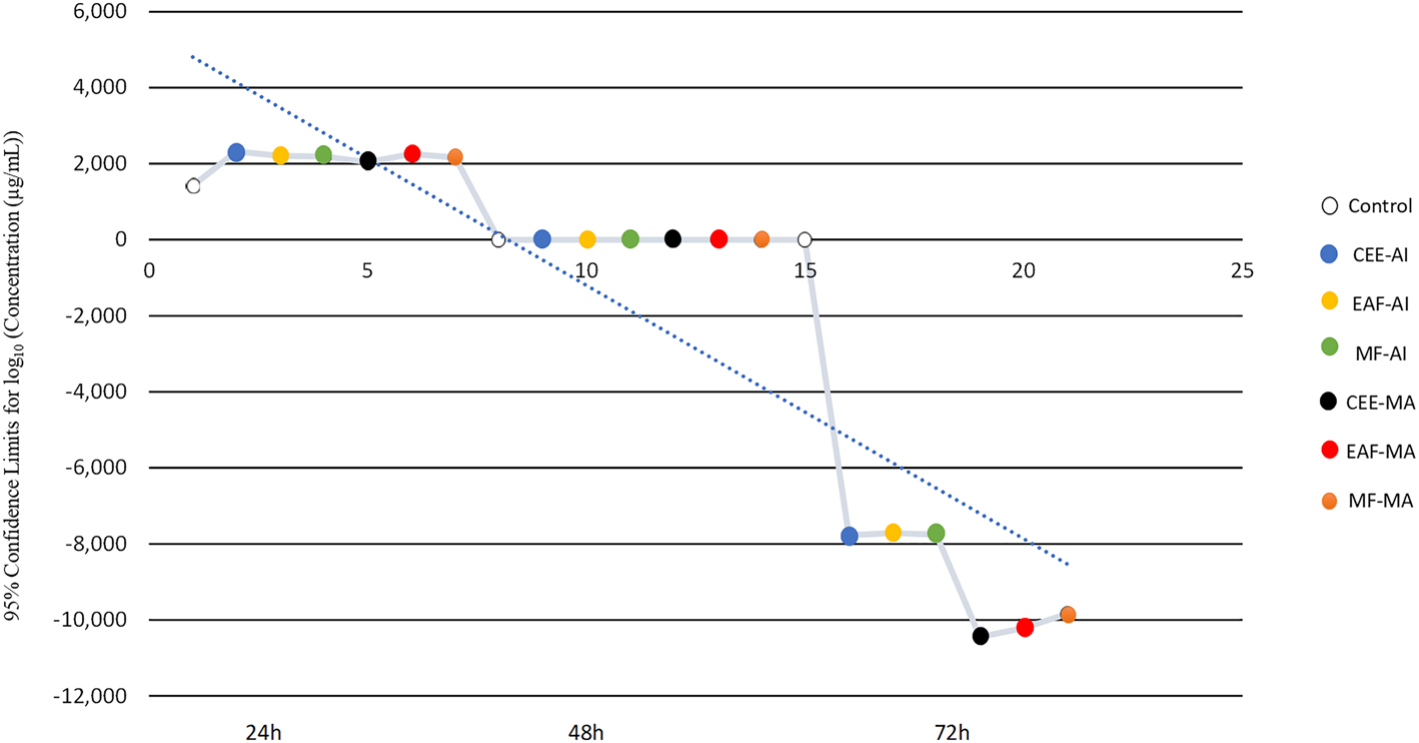
Estimate visualized in log10 with a reliability limit of 95% (Concentration (µg/mL)) in which can be observed the dependent variable “y” (concentration) as a function of “x” (time). The longer the concentrations of extracts of *Azadirachta indica. and Melia azedarach* and are in contact with *Artemia Salina*, the greater the toxicity level, thus reducing the number of living crustaceans in the study. CEE-AI: crude ethanolic extract—*Azadirachta indica*; EAF-AI: ethyl acetate fraction—*Azadirachta indica*; MF-AI: fraction methanol—*Azadirachta indica*; CEE-MA: crude ethanolic extract—*Melia azedarach*; EAF-MA: ethyl acetate fraction—*Melia azedarach*; MF-MA: fraction methanol—*Melia azedarach.*

The linear regression obtained using the probit test is shown in Fig. 1, which shows an estimate in log10 with a reliability limit of 95% [Concentration (µg/mL)], which can be observed as the dependent variable “y” (concentration) as a function of “x” (time). The longer the concentrations of extracts of *A. indica* and *M. azedarach* were in contact with *A. salina*, the greater the toxicity level, thus reducing the number of living crustaceans in the study and increasing the toxicity and decreasing the estimated dose of the LC_50_.

### Cell viability analysis using the MTT test (3-4,5-dimethyl-thiazol-2-yl-2,5-diphenyltetrazolium bromide)

**Figure 2:**
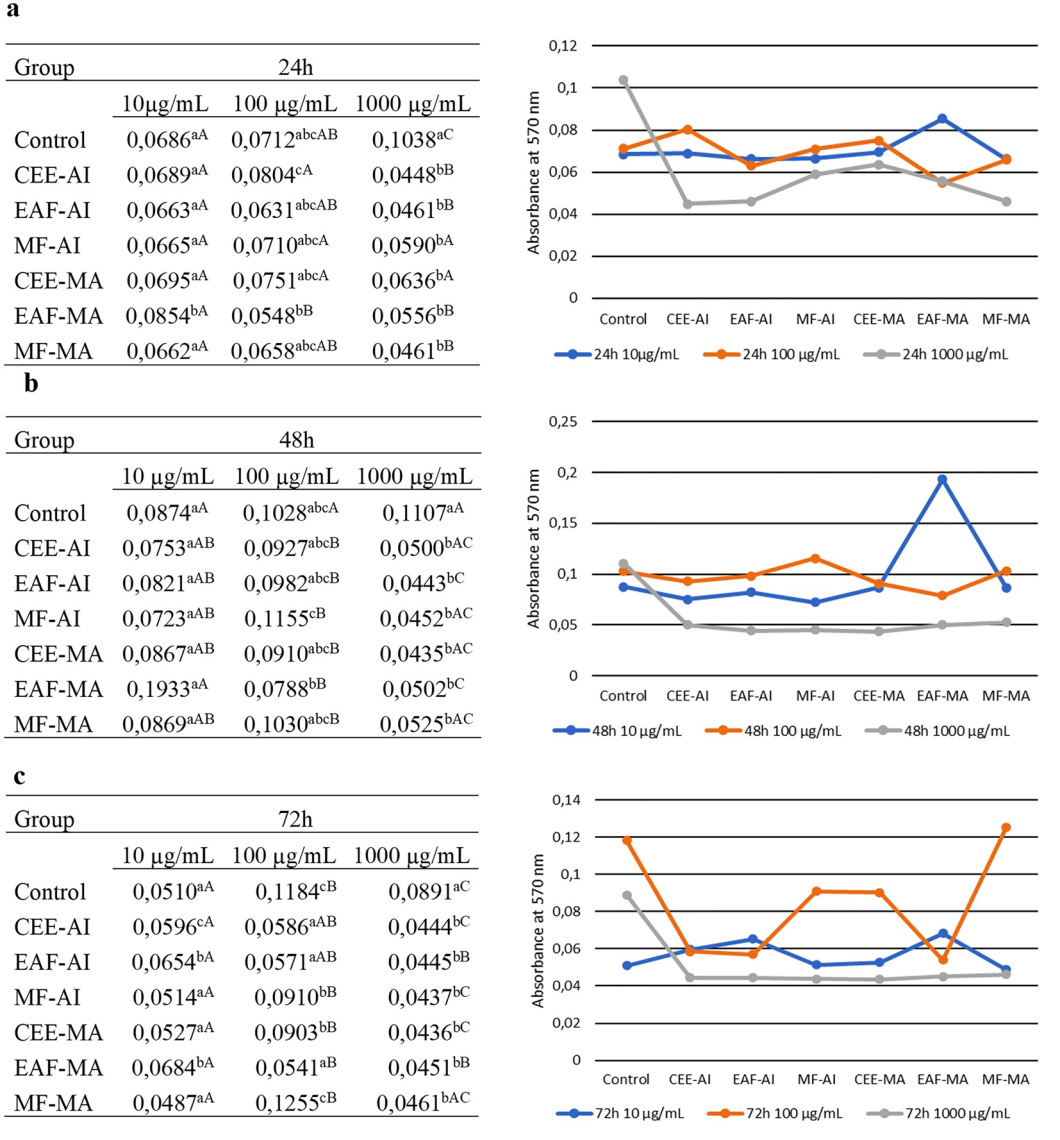
Tabulated and graphed values of absorbance (570 nm) obtained by the 3–4,5-dimethyl-thiazol-2-yl-2,5-diphenyltetrazolium bromide (MTT) test after the action of crude extracts of *Azadirachta indica* and *Melia azedarach* and their respective fractions of ethyl acetate and methanol for 24 h, 48 h, and 72 h. (**a**) treatment with the extracts and their respective fractions at concentrations of 10, 100, and 1000 µg/mL within 24 h; (**b**) treatment with the extracts and their respective fractions at concentrations of 10, 100, and 1000 µg/mL within 48 h; (**c**) treatment with the extracts and their respective fractions at concentrations of 10, 100, and 1000 µg/mL within 72 h CEE-AI: crude ethanolic extract—*Azadirachta indica*; EAF-AI: ethyl acetate fraction—*Azadirachta indica*; MF-AI: fraction methanol—*Azadirachta indica*; CEE-MA: crude ethanolic extract—*Melia azedarach*; EAF-MA: ethyl acetate fraction—*Melia azedarach*; MF-MA: fraction methanol—*Melia azedarach.*

The results of cell viability tests are shown in Fig. 2. At 24 h, the concentration of 10 µg/mL did not significantly differ between most treatments and controls for both plants, except for EAF-MA, which had a higher absorbance value than the others, with an approximate difference of 0.0103 nm (P < 0.05; P = 0.04). At a concentration of 100 µg/mL, the treatments Control, EAF-AI, MF-AI, CEE-MA, and MF-MA did not differ statistically among themselves; only treatments CEE-AI (P < 0.05; P = 0.01) and EAF-MA (P < 0.05; P = 0.01) differed from the others and between them. At 1000 µg/mL, all treatments were similar, except for the control group, with an approximate difference of 0.0402 nm (P < 0.05; P = 0.004). Among the concentrations of 10, 100, and 1000 µg/mL, at this same time (24 h), only the MF-AI and CEE-MA treatments were statistically equal, whereas the others were different (P < 0.05; P ≤ 0.004). In the control, CEE-AI, EAF-AI, and MF-MA treatments, the results were similar between the concentrations of 10 and 100 µg/mL and differed at 1000 µg/mL (P < 0.05; P ≤ 0.005). This indicated that all fractions from both plants, at a dosage of 1000 µg/mL, inhibited cell proliferation in the 24 h period compared to the control (Fig. 2a).

At 48 h, the 10 µg/mL concentration was statistically similar for all treatments. At a concentration of 100 µg/mL, the Control, CEE-AI, EAF-AI, CEE-MA, EAF-MA, and MF-MA groups were similar, and MF-AI treatment differed from EAF-MA (P < 0.05; P = 0.009). However, EAF-MA was similar to the control group and other treatments. At 1000 µg/mL, all treatments were identical and distinct from the control group (P < 0.05; P = 0.001). At concentrations of 10 µg/mL, 100 µg/mL, and 1000 µg/mL, at the same time, they were identical to each other in the control group. EAF-MA differed significantly among the three concentrations (P < 0.05; P = 0.001). The groups CEE-AI, EAF-AI, MF-AI, CEE-MA, and MF-MA obtained similar results, comparing 10 and 100 µg/mL. The concentration of 1000 µg/mL, on the other hand, was similar to 10 µg/mL in CEE-AI, MF-AI, CEE-MA, and EAF-AI treatments with identical results compared to 10 and 100 µg/mL, but different from 1000 µg/mL (P < 0.05; P = 0.002). Thus, all Meliaceae fractions studied at a concentration of 1000 µg/mL induced cell apoptosis within 48 h compared with the control. Furthermore, the EAF-MA fraction was inhibited at a dose of 100 µg/mL at 48 h (Fig. 2b).

At a time of 72 h, at a concentration of 10 µg/mL, the MF-AI, CEE-MA, and MF-MA treatments were similar to the control group, whereas the EAF-AI and EAF-MA treatments were similar to each other but differed significantly (P < 0.05; P = 0.003). CEE-AI showed a difference in all treatments, including the control group (P < 0.05; P ≤ 0.014). At a concentration of 100 µg/mL, only MF-MA was similar to that of the control group. The treatments MF-AI and CEE-MA were identical to each other and differed from the others, as were CEEI-AI, EAF-AI, and EAF-MA (P < 0.05; P ≤ 0.003). At a concentration of 1000 µg/mL, all treatments differed from the control group (P < 0.05; P ≤ 0.001) but were similar between them. Between the concentrations of 10, 100, and 1000 µg/mL, at the same time, the treatments Control, MF-AI, CEE-MA, EAF-MA, MF-MA were statistically different in all concentrations (P < 0.05; P ≤ 0.02). The CEE-AI and EAF-AI treatments were similar between 10 and 100 µg/mL and different at (P < 0.05; P 0.02) 1000 µg/mL. Thus, except for treatment with EAF-MA, which caused a reduction in cell multiplication at a concentration of 100 µg/mL, the other fractions showed this effect only at the highest dose (1000 µg/mL) of the control (Fig. 2c).

### Potential antiviral activity of leaf extracts of *Azadirachta indica* and *Melia azedarach*

Cell destruction and syncytium formation, which are cytopathic effects (CPE) typically caused by CLV, were observed in ovine third eyelid (OTE) cells during culture, even after treatment with both ethanolic leaf extracts. However, a gradual reduction in CPE was verified, which was directly proportional to the times (30, 60, and 90 min) in which phytocompounds were in contact with cells (Table 2).

**Table 2:** Levels of cytopathic effects in ovine third eyelid (OTE) cells after co-culture with somatic cells of goat colostrum and milk (CSC/MSC) treated with crude ethanolic extract (CEE) and the respective organic ethyl acetate (EAF) and methanol (MF) fractions of *Azadirachta indica* (AI) and *Melia azedarach* (MA) leaves.

Samples		Time (min.)	Viral cytopathic effects											
			Cell destruction						Presence of syncytium					
			C−	CEE	EAF	MF	C + T	C + P	C−	CEE	EAF	MF	C + T	C + P
Colostrum	AI	30	−	++++	+++	+++	+++	+++	−	+	+++	+++	+++	+++
		60	−	+++	++++	++++	++++	++++	−	+ +	+++	+++	++++	++++
		90	−	+	−	−	+	+ +	−	+ +	−	−	+++	+++
	MA	30	−	++++	++++	++++	++++	++++	−	+++	+++	+++	+++	++++
		60	−	+ +	+++	+++	+++	+++	−	+++	+ +	++	+++	+++
		90	−	−	−	+	++	++	−	−	−	++	++	++
Milk	AI	30	−	+	+	++	+++	+++	−	+++	+++	+++	++++	++++
		60	−	+	+	++	++++	++++	−	+++	+++	+++	+++	++++
		90	−	++	−	++	++	++	−	++	−	++	+++	+++
	MA	30	−	++	+	+	++++	++++	−	++	++	++	+++	+++
		60	−	−	−	+	+++	+++	−	−	−	++	+++	++++
		90	−	−	−	+	++	++	−	−	−	+	+++	++++

C−: negative control (only OTE cells); C + T.: positive control for the treatment (OTE and SCC or SMC cells infected with CLV strain—CAEV_CO_ without the addition of extracts), C + P.: positive slide control (OTE cells infected with CLV strain—CAEV_CO_); −: absence of cytopathic effects; +: very light effects; ++: light effects; +++: moderate effects; ++++: intense effects.

Significant destruction of cellular monolayers and moderate presence of syncytia occurred in colostrum samples submitted for 30 min to crude extracts of *A. indica* and* M*. *azedarach* and their respective ethyl acetate and methanol fractions. These CPEs were constant in the treatments for 60 min for the three *A. indica* extracts. However, a slight reduction in CPE was observed during the same period for all tested *M. azedarach* fractions. At 90 min, all extracts caused a greater reduction in CPEs.

For milk samples, the three fractions tested for 30 min yielded a slight reduction in cell destruction with a moderate presence of syncytia. In the 60 min treatment, CPE results of all *A. indica* extracts were similar to those of the previous period. In contrast, no CPE was observed after treatment with the crude extract and ethyl acetate fraction of *M. azedarach* for 60 min. After 90 min, only samples submitted to the ethyl acetate fraction of *A. indica* presented CPE, while in the remainder, and with respect to *M. azedarach*, only in the methanolic fraction, a slight formation of CPEs characteristic of goat lentivirus was observed.

**Figure 3:**
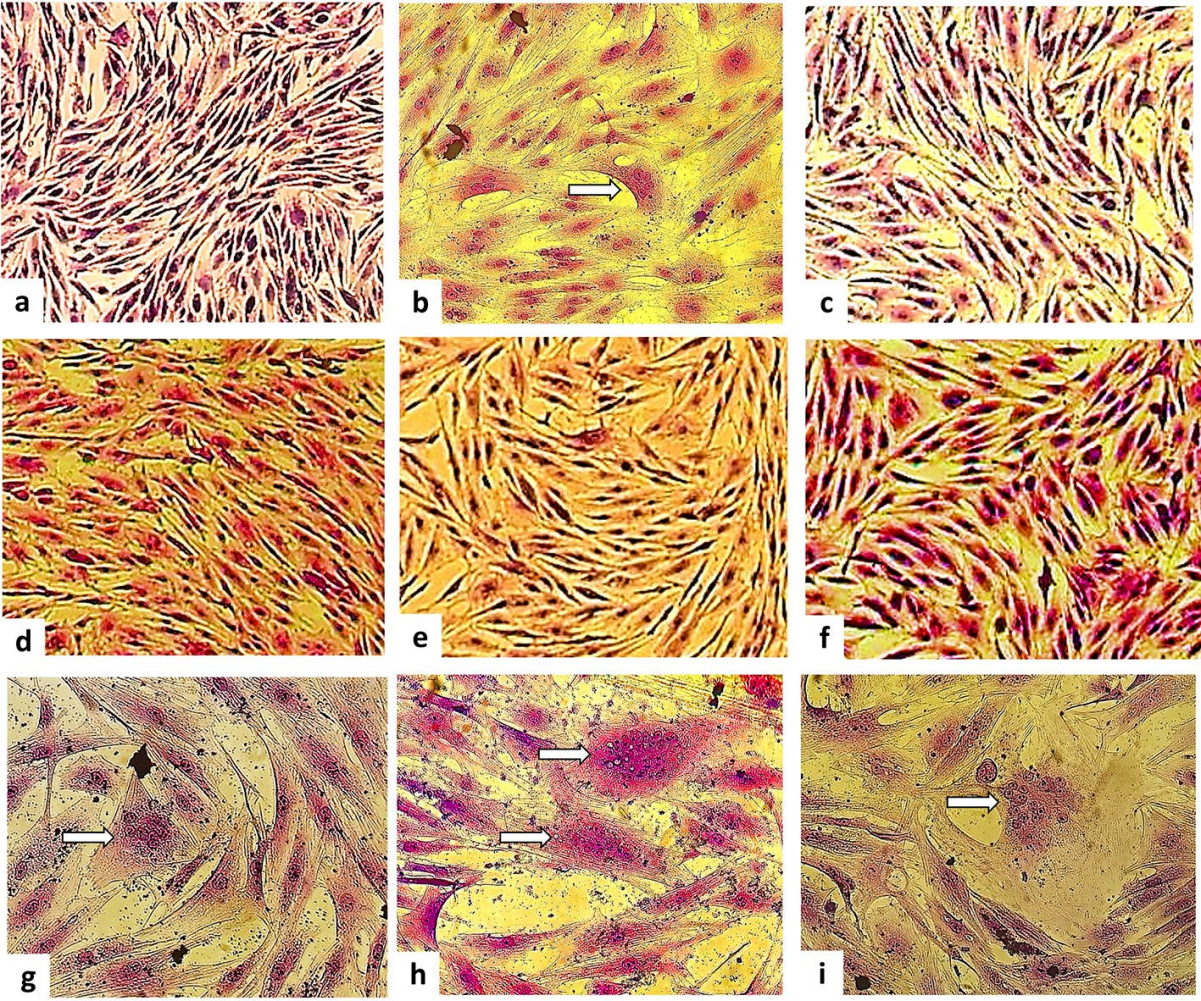
Co-culture with ovine third eyelid cells (OTE) and somatic cells from colostrum infected with CAEV_CO_ (SCC+) treated with *Azadirachta indica* and *Melia azedarach* leaf extracts for 90 min after 63 days of culture. (**a**) Negative culture control with OTE cells (×100 magnification). (**b**) Co-culture of OTE and SC*C*+ cells submitted to CEE*-AI* treatment*,* with syncytia (arrows) (×100 magnification). (**c**) Co-culture of OTE and SCC+ cells subjected to EAF-AI treatment (×100 magnification). (**d**) Co-culture of OTE and SC*C*+ cells subjected to MF-AI treatment (×100 magnification). (**e**) Co-culture of OTE and SC*C*+ cells submitted to CEE-MA treatment (×100 magnification). (**f**) Co-culture of OTE and SCC+ cells subjected to EAF-MA treatment (×100 magnification). (**g**) Co-culture of OTE and SCC+ cells subjected to MF-MA treatment (×200 magnification). (**h**) Co-culture of OTE and SC*C*+ with syncytia (arrows) (control treatment without extract) (×200 magnification). (**i**) Positive control of CAEV_CO_-infected OTE cells with syncytia (arrows) (×200 magnification). CEE-AI: crude ethanolic extract—*Azadirachta indica*; EAF-AI: ethyl acetate fraction—*Azadirachta indica*; MF-AI: fraction methanol *Azadirachta indica*; CEE-MA: crude ethanolic extract—*Melia azedarach*; EAF-MA: ethyl acetate fraction—*Melia azedarach*; MF-MA: fraction methanol—*Melia azedarach.*

These results demonstrated that the efficacy of both Meliaceae plants against CLV was more significant in the 90 min treatments for both colostrum and milk. In tests with colostrum, there was initially a cell culture of OTE, which represented the negative control culture, without evidence of CPE (Fig. 3a). Low levels of cell destruction and syncytium formation were observed in OTE cells treated with the crude ethanolic extract of *A. indica* (Fig. 3b, arrow). Nonetheless, no typical CLV CPE was observed with the crude extract of *M. azedarach* (Fig. 3e). The ethyl acetate fractions from both plants did not exhibit typical CPE (Fig. 3c,f). Furthermore, the methanol fraction of *A. indica* eliminated CPE entirely (Fig. 3d). However, syncytium formation was observed in the methanol fraction of *M. azedarach* (Fig. 3g, arrows). Regarding the positive controls of the tests, colostrum without previous treatment and culture with the standard CAEV_CO_ strain are shown in Fig. 3, in items 1h and 1i, respectively.

**Figure 4:**
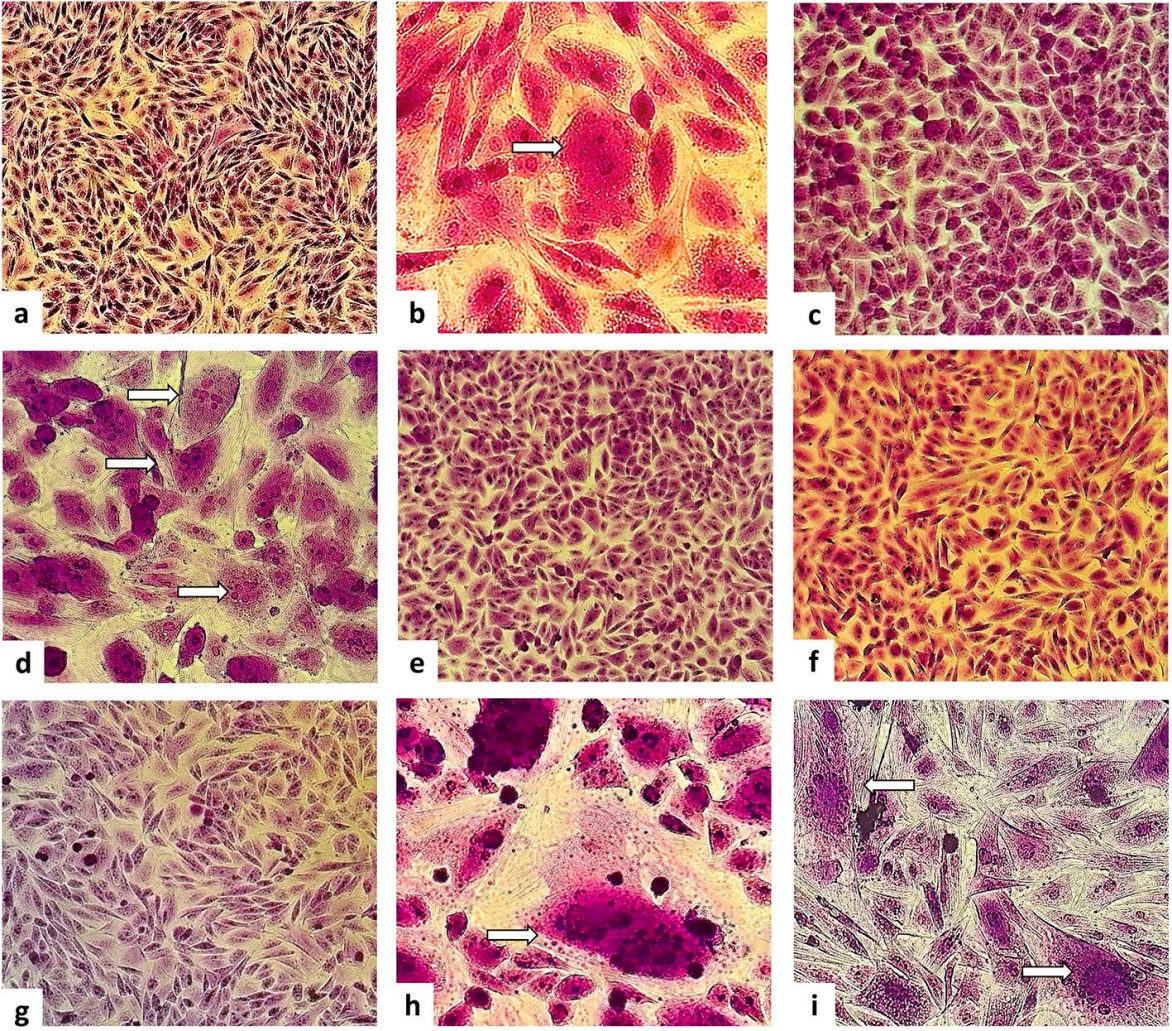
Co-culture with ovine third eyelid cells (OTE) and somatic cells from milk infected with CAEV_CO_ (SCM+) treated with *Azadirachta indica* and *Melia azedarach* leaf extracts for 90 min after 63 days of culture. (**a**) Negative culture control with OTE cells (×100 magnification). (**b**) Co-culture of OTE and SCM+ cells submitted to CEE-AI treatment*,* with syncytia (arrows) (×200 magnification). (**c**) Co-culture of OTE and SCM+ cells subjected to EAF-AI treatment (×100 magnification). (**d**) Co-culture of OTE and SCM + cells subjected to MF-AI treatment, with syncytia (arrows) (×100 magnification). (**e**) Co-culture of OTE and SCM+ cells submitted to CEE-MA treatment (×100 magnification). (**f**) Co-culture of OTE and SCM+ cells subjected to EAF-MA treatment (×100 magnification). (**g**) Co-culture of OTE and SCM+ cells subjected to MF-MA treatment (×100 magnification). (**h**) Co-culture of OTE and SCM+ with syncytia (arrows) (control treatment without extract) (×200 magnification). (**i**) Positive control of CAEVCO-infected OTE cells with syncytia (arrows) (×200 magnification). CEE-AI: crude ethanolic extract—*Azadirachta indica*; EAF-AI: ethyl acetate fraction—*Azadirachta indica*; MF-AI: fraction methanol *Azadirachta indica*; CEE-MA: crude ethanolic extract—*Melia azedarach*; EAF-MA: ethyl acetate fraction—*Melia azedarach*; MF-MA: fraction methanol—*Melia azedarach*.

The antiviral activity of the extracts was also observed in milk samples treated with both plants for 90 min. The Fig. 4a shows the negative control cells without any identified viral effects. In the treatment with *A. indica*, light levels of CPEs were observed, which were identified by the presence of syncytia in the culture using crude CEE and MF, respectively (Fig. 4b,d—arrows). Only the EAF of this plant completely inhibited the occurrence of typical CLV cytopathic effects (Fig. 4c). Concerning *M. azedarach* treatments, with the use of the crude extract and ethyl acetate fraction, CPE characteristics for goat lentivirus were not identified, and very light CPE was observed in the methanolic fraction (Fig. 4e–g). These data indicate the antiretroviral potential of the fractions that inhibited CPE formation in the co-culture. Lastly, the positive controls of the tests, colostrum without previous treatment, and culture with the standard CAEV_Co_ strain are shown in Fig. 4, in items 2h and 2i, respectively.

### Viral titration of samples

**Figure 5:**
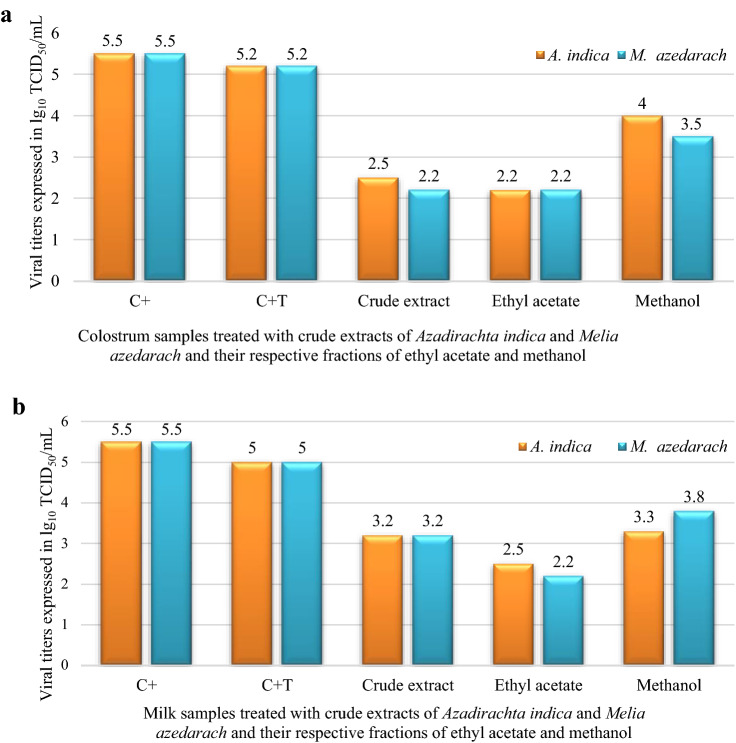
Viral titers of milk and colostrum samples co-cultured with ovine third eyelid (OTE) cells submitted to 90 min treatment composed of ethanolic leaf extracts of *Azadirachta indica* and *Melia azedarach*. (**a**) Colostrum samples treated with crude extract of *A. indica* and *M. azedarach* leaves and respective ethyl acetate and methanol fractions. (**b**) Milk samples treated with crude extract of *A. indica* and *M. azedarach* leaves and respective ethyl acetate and methanol fractions. *TCID50/mL: tissue culture infection dose is the highest dilution that presented, at 14 days post-inoculation, syncytia in 50% of inoculated wells, C+: positive control of standard CAEV_CO_ strain, C + T.: positive control of treatments.

The evidence found in the presence or absence of cytopathic effects typical of CLV in treatments with leaf extracts of *A. indica* and *M. azedarach* was confirmed by viral titration. A decrease in the number of infectious viral particles in the cell suspension of milk and colostrum samples subjected to 90 min of treatment with ethanolic extracts of the plants was observed in comparison to the controls (Fig. 5).

In goat infected colostrum samples (C + T), the initial viral titer was 10^5.2^ TCID_50_/mL, which was close to the result observed in the positive control of CAEV_CO_ (C+), 10^5.5^ TCID_50_/mL. After treatment with plant extracts, decreases in viral titers were observed to 10^2.5^, 10^2.2^ and 10^4^ TCID_50_/mL in samples treated with crude extract, ethyl acetate fraction, and methanol fraction of *A. indica*, respectively (Fig. 5a). Extracts of *M. azedarach* yielded similar results, which were 10^2.2^, 10^2.2^, 10^3.5^ TCID_50_/mL for crude extract, ethyl acetate fraction and methanol fraction, respectively. Furthermore, the ethyl acetate fraction presented the best results in both tests in comparison to the C + T control with a logarithmic difference of 3 lg between viral titers, demonstrating a relevant and promising antiviral potential.

In milk samples, the antiviral potential of ethyl acetate fractions of *A. indica* and *M. azedarach* were constant, presenting values of 10^2.5^ and 10^2.2^ TCID_50_/mL, respectively. These values also demonstrated that there was logarithmic difference of 2.3 lg and 2.8 lg, respectively, between titers in comparison to the positive milk control (C + T), which was 10^5^ TCID_50_/mL (Fig. 5b). Crude extracts and organic methanol fractions from both plants showed elevated titer values, 10^3.2^ and 10^3.2^ TCID_50_/mL for crude extract, and 10^3.3^ and 10^3.8^ TCID_50_/mL for methanol fraction of *A. indica* and *M. azedarach*, respectively. These values demonstrated lower efficacy than those from the ethyl acetate fractions of both plants.

Hence, 150 µg/mL concentration of ethyl acetate fractions of ethanolic extracts from *A. indica* and *M. azedarach* leaves could potentially reduce the viral titer of CLV in colostrum by a thousandfold. The inhibition in milk was 500–800 times for the respective plants.

### Somatic cell count (SCC) of colostrum and milk after treatment with extracts of *A. indica* and *M. azedarach*

From the data obtained in the co-culture and in the viral titration, it was observed that the 90-min treatment with the crude extracts of the leaves of *A. indica* and *M. azedarach* and their respective EFA and MF obtained the best antiviral responses. To determine the number of cells and, consequently, the quality of colostrum and milk, the somatic cell counts of the samples obtained after treatment with the extracts were determined.

**Figure 6:**
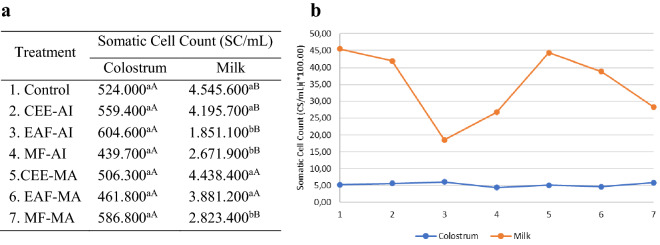
Tabulated values (**a**) and graphs (**b**) of somatic cell count (SCC) by the microscopic method in colostrum and goat milk samples after treatment with the crude extracts of *Azadirachta. indica* and *Melia azedarach* and the organic fractions of Ethyl Acetate and Methanol for 90 min of action. Different letters in the same column differ from each other by Tukey's test P < 0.01 CEE-AI: crude ethanolic extract—*Azadirachta indica*; EAF-AI: ethyl acetate fraction—*Azadirachta indica*; MF-AI: fraction methanol *Azadirachta indica*; CEE-MA: crude ethanolic extract—*Melia azedarach*; EAF-MA: ethyl acetate fraction—*Melia azedarach*; MF-MA: fraction methanol—*Melia azedarach*.

Figure 6 shows somatic cell count data after these treatments. SCC of colostrum samples did not differ statistically; the control treatment resulted in an average of 524.000 SC/mL (somatic cell/milliliters), and the other results were statistically similar to it, with P < 0.01. There was a significant difference in milk between the control sample, with an average of 4.545.600 SC/mL (P = 0.008), concerning the EAF-AI treatments, with an average of 1.851.100 SC/mL, and MF-AI, 2.671.900 SC/mL (P = 0.003). In the treatment with *M. azedarach* extracts, a statistical difference was detected only in the MF-MA, with a mean value of 2.823.400 CS/mL (P = 0.009), with P < 0.01. Thus, we can observe a reduction in SCC in the milk samples that had contact with the strata with the EAF-AI, MF-AI, and MF-MA treatments at a 150 µg/mL concentration.

### Chromatograms of* A. indica *and* M. azedarach* extracts

**Figure 7:**
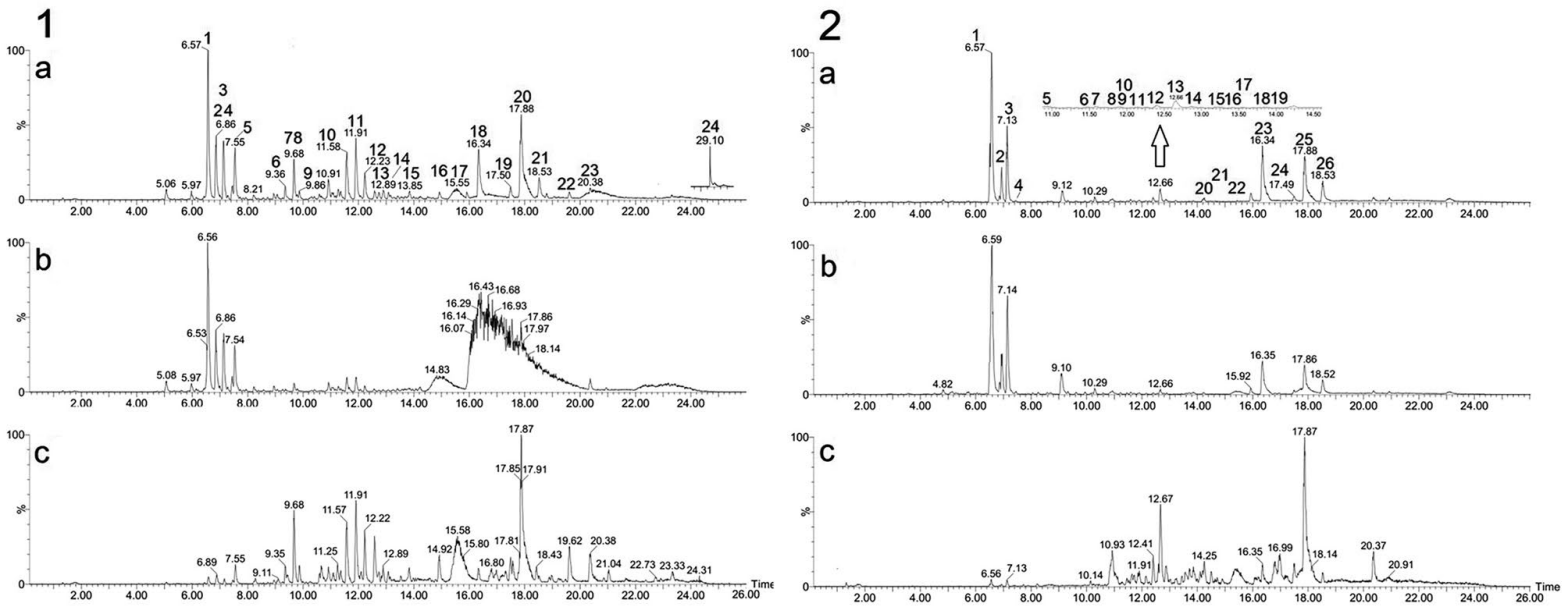
UPLC-ESI-QTOF-MS^E^ chromatograms of *Azadirachta indica* (**1**) and *Melia azedarach* (**2**) leaves extracts in negative ionization mode: (**a**) crude ethanol extract; (**b**) methanol fraction; (**c**) ethyl acetate. The numbers on the peaks are referenced in Tables 3 and 4.

The chromatograms for the ethyl acetate (a), methanol (b), and crude ethanol extract (c) fractions of *A. indica* leaves are shown in Fig. 7-1. In the ethyl acetate fraction (Fig. 7-1a), nimbandiolactone-23 isomer II (8), azedarachin C isomer I (10), isomer II (11), isomer III (12), flowerone (13), trisinlin A (15), licoflavanone (16), sulfoquinovosylmonoacylglycerol (SQMG) 18:3 (17), monogalactosylmonoacylglycerol (MGMG) 18:3 (20), melianone (22), and MGMG 16:0 (23). In the methanolic fraction (Fig. 7-1b) and crude ethanolic extract (Fig. 7-1c), rutin (1), quercetin-*O*-hexoside (2), kaempferol-*O*-rutinoside (3), kaempferol-*O*-hexoside (4), and quercetin-*O*-rhamnoside (5) were predominant.

**Table 3:** Compounds putatively identified in ethyl acetate fraction from *Azadirachta indica* leaves by UPLC-ESI-QTOF-MSE in negative ionization mode.

Peak	Rt (min)	[M−H]^−^ observed	MS^2^ ions	Molecular formula	Putative name (class)	ID level^a^	Ref.
1	6.58	609.1459	300.0174, 178.9952, 151.0022	C_27_H_30_O_16_	Rutin (flavonoid)	1	^1^
2	6.89	463.0882	300.0260, 271.0249, 255.0292, 178.9958, 151.0027	C_21_H_20_O_12_	Quercetin-*O*-hexoside (flavonoid)	1	^2^
3	7.16	593.1506	285.0388, 255.0250	C_27_H_30_O_15_	Kaempferol-*O*-rutinoside (flavonoid)	1	^3^
4	7.46	447.0922	285.0382, 284.0294, 255.0265, 151.0021	C_21_H_20_O_11_	Kaempferol-*O*-hexoside (flavonoid)	1	^3^
5	7.55	447.0920	300.0226, 271.0203, 255.0275, 178.9947, 151.0010	C_21_H_20_O_11_	Quercetin-*O*-rhamnoside (flavonoid)	1	^3^
6	9.35	487.1963	443.2097, 411.1812, 393.1742, 245.1179, 179.0708, 137.0597	C_26_H_32_O_9_	Nimbandiolactone-23 isomer I (triterpenoid)	3	^5^
7	9.43	301.0340	271.0175, 179.0720, 151.0006, 121.0262	C_15_H_10_O_7_	Quercetin (flavonoid)	1	^1,6^
8	9.68	487.1964	443.2092, 411.1808, 393.1728, 179.0695	C_26_H_32_O_9_	Nimbandiolactone-23 isomer II (triterpenoid)	3	^5^
9	9.86	487.1963	443.2055, 411.1879, 393.1808, 245.1171, 179.0684, 137.0573	C_26_H_32_O_9_	Nimbandiolactone-23 isomer III (triterpenoid)	3	^5^
10	11.57	585.2703	503.2330, 471.2082, 427.2124, 395.1866, 371.1883	C_32_H_42_O_10_	Azedarachin C isomer I (triterpenoid)	3	^7^
11	11.91	585.2700	503.2304, 427.2105, 371.1872, 353.1771	C_32_H_42_O_10_	Azedarachin C isomer II (triterpenoid)	3	^7^
12	12.22	585.2703	509.2555, 503.2299, 427.2122, 371.1858	C_32_H_42_O_10_	Azedarachin C isomer III (triterpenoid)	3	^7^
13	12.58	355.1170	329.2322, 283.0607, 151.0012	C_20_H_20_O_6_	Flowerone (flavonoid)	3	^8^
14	12.89	627.2809	565.3752, 469.2244, 427.2140, 297.2460, 81.0343	C_34_H_44_O_11_	Isosalanninolide (triterpenoid)	3	^1^
15	13.83	529.2806	479.2871, 443.2826, 401.2700, 297.2418, 116.9263	C_30_H_42_O_8_	Trisinlin A (triterpenoid)	3	^9^
16	14.92	339.1241	187.1077, 150.9989	C_20_H_20_O_5_	Licoflavanone (flavonoid)	1	^1,4^
17	15.58	577.2689	299.0433, 277.2194, 225.0041	C_27_H_46_O_11_S	Sulfoquinovosylmonoacylglycerol (SQMG 18:3) (glycolipids)	2	^10^
18	16.33	675.3588	415.1454, 397.1332, 305.0900, 277.2150	C_33_H_56_O_14_	Digalactosylmonoacylglycerol (DGMG 18:3) (glycolipids)	2	^4,11^
19	17.57	489.3580	407.1914, 399.2893, 219.0653, 187.1079	C_30_H_50_O_5_	Meliantriol (triterpenoid)	3	^1^
20	17.87	513.3057	277.2098, 253.0908, 235.0815	C_27_H_46_O_9_	Monogalactosylmonoacylglycerol (MGMG 18:3) (glycolipids)	2	^4,11^
21	18.53	653.3755	415.1475, 397.1361, 305.0917, 235.0816	C_31_H_58_O_14_	DGMG 18:0 (glycolipids)	2	^11^
22	19.62	469.3310	325.1862, 183.0092, 116.9283	C_30_H_46_O_4_	Melianone (triterpenoid)	3	^12^
23	20.38	491.3242	255.2299, 253.0922, 235.0826	C_25_H_48_O_9_	MGMG 16:0 (glycolipids)	2	^10^
24	29.09	459.2540	325.1821, 311.1630, 277.2139, 183.0033	C_30_H_36_O_4_	Unknown	4	–

^a^Identification level: 1. Exact mass, MS/MS (Mass Spectrometry) fragments and occurrence; 2. Exact mass and MS/MS fragments; 3. Exact mass and occurrence; 4. Not identified.

**Table 4:** Compounds putatively identified of extract in ethyl acetate fraction from *Melia azadirachta* leaves by UPLC-ESI-QTOF-MSE in negative ionization mode.

Peak	Rt (min)	[M−H]^−^	MS^2^ ions	Molecular formula	Putative name (class)	ID level^a^	Ref.
1	6.58	609.1456	301.0375, 300.0287	C_27_H_30_O_16_	Rutin (flavonoid)	1	^1^
2	6.89	463.0882	301.0345, 300.0430	C_21_H_20_O_12_	Quercetin-*O*-hexoside (flavonoid)	1	^2^
3	7.16	593.1495	285.0417, 284.0413	C_27_H_30_O_15_	Kaempferol-*O*-rutinoside (flavonoid)	1	^3^
4	7.46	447.0947	285.0227	C_21_H_20_O_11_	Kaempferol-*O*-hexoside (flavonoid)	1	^3^
5	10.93	327.2179	229.1460, 211.1338	C_18_H_32_O_5_	9,12,13- Trihydroxyoctadeca- dienoic acid (fatty acid)	2	^4^
6	11.45	673.2863	575.2546	C_35_H_46_O_13_	Toosedane B isomer (triterpenoid)	1	^5^
7	11.52	615.2801	573.2682, 555.2617	C_33_H_44_O_11_	Meliazedalide A (triterpenoid)	1	^7^
8	11.64	329.2337	229.1462, 211.1313	C_18_H_34_O_5_	Trihydroxyoctadecaenoic acid isomer (fatty acid)	2	^4^
9	11.72	647.2856	615.2759, 603.2880	C_37_H_44_O_10_	23-methoxyohchininolide isomer (triterpenoid)	1	^8^
10	11.91	647.2864	615.2800, 603.2820	C_37_H_44_O_10_	23-methoxyohchininolide isomer (triterpenoid)	1	^8^
11	12.15	673.2846	575.2642	C_35_H_46_O_13_	Toosedane B isomer (triterpenoid)	1	^5^
12	12.41	715.2982	687.3081, 655.2804, 623.3573	C_37_H_48_O_14_	12-acetyltrichilin B (triterpenoid)	1	^9^
13	12.67	729.3122	669.2884, 637.2593	C_38_H_50_O_14_	Unknown	5	–
14	12.87	729.3154	669.2927, 625.3054, 593.2753	C_38_H_50_O_14_	Unknown	5	–
15	13.25	615.2981	–	C_37_H_44_O_8_	Trichilinin D (triterpenoid)	3	^8^
16	13.45	647.2845	615.2758, 603.2639	C_37_H_44_O_10_	23-methoxyohchininolide isomer (triterpenoid)	1	^8^
17	13.57	311.1860	293.1956, 267.1960	C_17_H_28_O_5_	Dihydroartemisinin ethyl ether	2	^9^
18	13.71	293.2110	–	C_18_H_30_O_3_	Hydroxylinolenic acid isomer (fatty acid)	4	^10^
19	13.86	293.2115	–	C_18_H_30_O_3_	Hydroxylinolenic acid isomer (fatty acid)	4	^10^
20	14.25	329.2325	229.1456, 211.1237	C_18_H_34_O_5_	Trihydroxyoctadecaenoic acid isomer (fatty acid)	2	^4^
21	14.51	845.2927	311.1942, 147.0447	C_52_H_46_O_11_	Unkown	5	–
22	15.44	577.2620	–	C_30_H_41_O_11_	Unkown	5	–
23	16.35	397.1459	–	C_26_H_21_O_4_	Unkown	5	–
24	16.99	275.2008	–	C_18_H_28_O_2_	Stearidonic acid (fatty acid)	4	^11^
25	17.89	277.2158	233.2280	C_18_H_30_O_2_	Linolenic acid (fatty acid)	1	^11,12^
26	20.37	255.2319	183.0182	C_16_H_32_O_2_	Palmitic acid (fatty acid)	2	^11,13^

^a^Identification level: 1. Exact mass, MS/MS (Mass Spectrometry) fragments and occurrence; 2. Exact mass and MS/MS fragments; 3. Exact mass and occurrence; 4, Exact mass; 5. Not identified.

With regard to *M. azedarach*, the chromatograms for the crude ethanolic extract (a), methanolic fraction (b) and ethyl acetate (c) are shown in Fig. 7-2. In the crude ethanol extract (Fig. 7-2a) and methanolic fraction (Fig. 7-2b), the flavonoids rutin (1), quercetin-O-hexoside (2), and kaempferol-O-rutinoside (3). In the ethyl acetate fraction (Fig. 7-2c), compounds 9,12,13-trihydroxyoctadeca-dienoic acid (6) and linolenic acid (26) stood out, in addition to the unknown compound 14.

### Chemical composition and characterization of *A. indica* and *M. azedarach* leaf extracts

The organic EAF from both plants showed a greater reduction in the viral titer of the CAEV_CO_ strain in the colostrum and milk samples, and consequently, a better antiviral action. Thus, in negative ionization mode, their chemical compositions were determined by ultra-performance liquid chromatography-mass spectrometry (UPLC/MS). In the fraction derived from *A. indica*, 24 compounds were identified, of which eight were flavonoids (1–5, 7, 13, and 16), ten were triterpenoids (limonoids-type—6, 8–12, 14, 15, 19 and tirucalane-type–22), five were glycolipids (17, 18, 20, 21, and 23), and one was an unknown compound (24) (Table 3). In the chemical composition of ethyl acetate from *M. azedarach*, 27 compounds were determined, of which four were flavonoids (1–3 and 5), eight were fatty acids (6, 9, 19–21 and 25–27), eight were triterpenoids (limonoids-type—7, 8, 10–13, 16, and 17), a monoterpene lactone (4), a sesquiterpene (18), and five unknown compounds (14, 15, and 22–24) (Table 4).

## Discussion

Much research has been conducted on bioactive substances to investigate their possible antiviral effects against infections in humans and animals. In this context, plants from several botanical families, and their parts, such as the leaves, roots, flowers, and seeds, have been studied and revealed to possess promising substances in their composition^27^.

From bibliographic research, it was shown that potential antiviral effects with species of Meliaceae were achieved at an approximate concentration of 150 µg/mL^28^. From this, pilot tests were carried out with this concentration, which showed promising results; therefore, it was decided to elucidate its possible antiviral effect against SRLV in more detail.

Countless researchers use tests with *A. salina* Leach as an effective bioassay for the elucidation of lethal dosages of bioactive components present in natural products that are screened for different purposes^29–32^, owing to their speed, low cost, and reliability of results^29^. In general, a natural product is considered toxic when its lethal concentration is less than 1000 µg/mL, within 24 h^29^. It is noteworthy that this 24-h period is standard in several studies^29,31–33^. However, in the present study, toxicity was observed after 48 h of incubation in all treatments. Thus, the results of the toxicity test with *A. salina* Leach demonstrated that a dosage of 150 µg/mL is a safe margin for using extracts of both Meliaceae in 24 h, with no toxic effect observed. Although the CEE-MA and MF-MA treatments obtained LC_50_ lower than the test concentration, they remained within the maximum limits for use of these extracts during the study period.

The toxic effects of some extracts are often directly linked to solvents^33^. In the present study, we chose to use dimethylsulfoxide (DMSO) because it is highly soluble and does not cause molecular interference in the phytochemical components^34^, unlike Tween 80, which is reported to be more toxic than other solvents through causing protein denaturation and inhibition of physiological processes^35,36^. In determining the LC_50_ of NaCl, it was shown that the test dosage, which has already been adopted in previous studies^30,37,38^, was lower than the dose considered lethal in this study, thus not exerting any effect on the outcomes of no treatment.

In the evaluation of the viability of OTE cells via the MTT test, it was shown that the extracts of both Meliaceae and their respective fractions at the dosage tested did not cause toxicity to this cell type, and there was no inhibition of cell proliferation. Interestingly, in the literature, there are reports of cytotoxicity of some species of Meliaceae that, when used at high doses (1 mg/mL), induced cell death by apoptosis at 24 and 48 h, while lower concentrations (10 µg/mL and 100 µg/mL) did not show a reduction in cell viability^39^ as in the present study.

The use of cell viability assays is always an effective way to determine biological toxicity when working with plant extracts, and MTT, in general, is the method of choice for in vitro evaluations to detect the harmful effects of natural products^40^ in the most distinct cell types. In this study, we chose to use nictitating membrane cells because of the ease of obtaining them, the absence of animal sacrifice^41^, and because it was used throughout this antiviral experiment. Thus, this cell type is not widespread, making it difficult to provide data in the literature that can serve as comparative parameters.

Hence, the data obtained in this study performed with co-culture of SC of colostrum and SC of milk and OTE cells after use of ethanolic extracts of *A. indica* and *M. azedarach* may reveal a possible antiviral activity against CLV. This fact was observed mainly in the organic fraction of ethyl acetate, with an action time of 90 min, with marked inhibition of syncytium formation and cell destruction, which are considered the main characteristic cytopathic effects of CVL elucidated even in previous research studies, where the adoption of a qualitative evaluation through the visualization of their occurrence levels in cultured cells is standard^19,42–46^.

Thus, the virulence of positive colostrum and milk samples for CLV was affected by treatments with leaf extracts of *A. indica* and *M. azedarach,* as observed in co-culture, since co-culture has several applications in biology and in the study of natural or synthetic interactions among cell populations. This technique may be defined as a variation in cell culture, in which two or more different cell populations are incubated with some degree of contact between them^47^. Therefore, free viral transmission may occur in the extracellular environment or through cell-to-cell interactions, involving direct contact between them^48^.

Concerning antiviral activity, the reported findings consolidate the use of these Meliaceae species as promising herbal medicines. Antiviral activity of *A. indica* has been reported against coxsackievirus (group B) using ketone extracts^49^, and against dengue virus using aqueous extracts^28^ and specific isolates, such as triterpenoids^50^. HIV is also inhibited by neem when administered via intravaginal tablets in women, as demonstrated by studies in India performed in vivo^25^ and in vitro^51^ with CD4+ cells. This plant has also been tested against papillomavirus type 1 (PV-1)^52^ and herpes simplex virus (HSV)^53^ using polysaccharides. In addition, aqueous extracts of this herbal medicine have been shown to be effective against foot-and-mouth disease^24,54^. Furthermore, flavonoids from this species of Meliaceae inhibited the viral activity of several influenza strains (H_1_N_1_, H_1_N_2_, H_2_N_2_, H_2_N_3_, H_5_N_1_, H_7_N_2_, H_7_N_3_, H_7_N_7_ and H_9_N_2_)^55^.

Basic studies performed with extracts of *M. azedarach* reported antiviral activity against Sindbis virus^56^. In 1998, Castilla et al.^57^ inhibited the multiplication of the Junin virus using meliacine peptide. More recent findings have demonstrated that extracts from chinaberry trees affect DNA synthesis, maturation, and egress of HSV-1^58,59^ and inhibit viral multiplication of HSV-2^26^. In addition, fruit extracts of *M. azedarach* inactivated dengue virus, yellow fever virus, and West Nile virus^60^*.* Furthermore, strains of the influenza virus (H_1_N_1_, H_3_N_2_, H_5_N_1_, H_7_N_9_ and H_9_N_2_)^23^ and HSV-3^61^ were inhibited by aqueous and ethanolic extracts of the same plant, respectively.

The viral titration method used in this study after co-culture investigated the antiviral activity of ethanolic leaf extracts of *A. indica* and *M. azedarach* in colostrum and milk samples infected with CLV. The use of these plants reduced CLV titers, which means a decreased number of viral particles in the cell supernatant, in the order of 1000 times in colostrum and 800 times in milk. However, complete elimination of CLV did not occur in these samples. However, these plants may be considered efficient herbal medicines for antiretroviral control. Similar data was reported in research by Narovlianskiĭ et al.^62^*,* which studied antiviral effects of sodium polyprenyl phosphate (PPP) against hepatitis C virus (HCV) in swine embryo kidney cells (SPEV) and demonstrated that 60 µg of PPP decreased HCV titers with a 3.5 lg difference.

Moreover, the more intense reduction in viral titers in colostrum samples may be attributed to the interaction of secondary metabolites with proteins that naturally occur more frequently in this type of biological sample. One example is lactoferrin, a glycoprotein that has variable concentrations according to the animal species^63^ and contributes to the development of the immune system^64^. This protein has been reported to be effective against rotavirus^65^ because of its possible role in stopping the entry of this pathogen into host cells through blocking viral receptors or via direct ligation with viral particles^66^. This fact may have been potentiated by the synergistic activity of components found in ethanolic leaf extracts of the investigated plants,and may have favored an improved antiretroviral activity of fractions in goat colostrum, considering that flavonoids are capable of interacting with lactoferrin in the secretions of ruminants^67^.

In this study, a higher concentration of distinct ethanolic fractions from both Meliaceae plants was used in comparison with other studies. Values of 12.1 to 80 µg/mL of *A. indica* were effective against PV-1^52^, while 63.5 µg/mL of *M. azedarach* was efficient in inhibiting herpes simplex virus type 1 (HSV-1)^59^. However, the effective dose of a substance against a strain varies according to viral type. This occurs because of the high capacity of mutation of viruses and the necessity for greater concentrations of substances for satisfactory inhibitory effects against viral agents^19,24,68^.

There are reports that the number of somatic cells (macrophages, lymphocytes, neutrophils, epithelial desquamation cells, etc.) may be related to the presence of proviruses in the samples, mainly in macrophages^12,13^. Thus, in this study, there was a reduction in the viral titer after treatment with the extracts of both Meliaceae, showing that all colostrum samples had SCC similar to the control and within the standards established for the species^69^, the extracts in this biological sample had a potent antiviral effect. Furthermore, in milk, the reduction of SCC due to the action of some treatments (EAF-AI, MF-AI, and MF-MA) is likely to be associated with this drop in the viral titer attributed to the antiviral effect of the extracts since the referred fractions where this effect was observed showed lower SCC. In general, high numbers of somatic cells are indicative of caprine lentivirus infection, as values > 1,000,000 cells/mL are considered evidence of subclinical mastitis in goats infected with this viral agent^70–72^. Thus, the reduction in somatic cells in some samples may be associated with a reduction in viral particles caused by the Meliaceae tested, which consequently led to a reduction in viral multiplication.

It is worth noting that information on SCC in goat colostrum is scarce^73^, and Brazilian legislation still requires a certain number of SC in goat milk^74^. In addition, numerous variables (species, level of infection, physiological state, and management practices) influence the SCC in milk^75^. Although there have been attempts to establish SCC in non-infected goats, biological and instrumental limitations make it difficult to compare results and establish this parameter in goats^74^. However, with SCC in the treatments in which there was a reduction, the amount was still within the established limits (1,000,000 SC/mL) by some authors referring to this parameter for small ruminants^69,74^.

Chemical analysis of the different fractions used in this study revealed a composition of phenols, flavonones, flavonols, xanthones, steroids, flavonoids, triterpenoids, saponins, and alkaloids. These components are usually reported in plants from the Meliaceae family^59^, and the antiviral effect is attributed to secondary metabolites that may act alone or synergistically^76–78^. Furthermore, when acting in consonance, these substances present great pharmacological variability and may, in the future, be used as broad-spectrum antimicrobial agents^79,80^.

In terms of phytochemical composition, in several other studies, with the exception of glycolipids (17, 18, 20, 21, and 23), the other compounds were previously reported in *A. indica* (1–9, 13, 14, 16, and 19)^55,81–84^ and in the Meliaceae family (10–12, 15, 22)^67,85–87^. In *M. azedarach*, with the exception of fatty acids (6, 9, 19–21 and 25) and unknown compounds (14, 15 and 22–24), the other compounds have been previously reported (1–3, 5, 7, 8, 10–13, 16–18, 26, and 27)^67,83–87^. Thus, it is suggested that limonoid-type flavonoids (6, 8–12, 14, 15, and 19), tirucalane-type (22), and glycolipids (17, 18, 20, 21, and 23) are the compounds that determine the antiviral activity of the ethyl acetate fraction of *A. indica*. In *M. azedarach*, limonoid-type compounds (7, 8, 10–13, 16, and 17) determine the antiviral activity of the ethyl acetate fraction.

In addition, the vast majority of phytocompounds with antiviral activity are derived from aqueous and ethanolic extracts^23,24,28,54,60,61^. The study of pharmacological applications of plant components, such as alkaloids, flavonoids, and terpenoids, is fundamental in researching efficient antivirals. Moreover, flavonoids are bioactive substances with antiviral activity in isolated preparations or distinct vegetable extracts^55,77,88,89^. These phytocompounds may act in the inhibition of proteases and enzymes, such as reverse transcriptase^55,89^, which is responsible for catalyzing the reverse transcription of viral RNA (ribonucleic acid) in dsDNA (double-stranded deoxyribonucleic acid) molecules that may be integrated into the host cell genome^90^.

Although they are found in all polarities, some authors have identified and reported that they are common for secondary metabolites (flavonoids, triterpenoids, etc.)^91,92^. This fact corroborates the present study, in which treatment with the ethyl acetate fraction of both plants resulted in a reduction in cytopathic effects and a reduction in the viral titer of the CAEV_CO_ strain, indicating a potential antiviral effect.

Among the 300 chemically active compounds isolated from *A. indica*^79^, most belong to the flavonoid class, as Meliaceae is one of the few species that produces them in such abundance^93^, which are attributed to its vast biological and physiological activities^79,94^. One of the main flavonoids evidenced in phytochemical analyses in *A. indica* is quercetin, which is often found in extracts from this plant. and which in general, quercetin is involved in the mechanism of action of the most distinct pharmacological properties^92–96^, including antiviral^97^, against hepatitis C virus^98^, herpesvirus^99^, H_1_N_1_^100^, and coronavirus^101^. Quercetin has promising antiviral effects via inhibition of proteases, suppression of protein receptors on the viral capsid, and blockade of reverse transcriptase^102–104^. It is noteworthy that SRLV requires reverse transcriptase to transcribe viral RNA into proviral DNA, which is integrated into the host cell genome via an integrase enzyme, a primordial step throughout the viral replication cycle^90^. Thus, the bioactive compound that acts in this process may play a valuable role in combating these viral agents.

In addition to flavonoids, triterpenoids were also present in the fractions of both Meliaceae species. In the case of *M. azedarach*, the triterpenoids had a greater number of representatives than the flavonoid classes in the fraction that had the best antiviral activity against SRLV. These phytoconstituents, such as quercetin, are the targets of investigation in the search for bioactive components with the most distinct pharmacological properties^105^. There are reports of antiviral activity attributed to triterpenoids present in vegetables against the dengue virus^50,106^, enterovirus 71^107^, coronavirus^108^, HIV, H_1_N_1_, H_5_N_1_ and HSV^109^. The mechanism by which triterpenoids exert their antiviral action mainly involves blocking the enzymes involved in the replicative process^109^.

Studies involving antiretroviral therapy have focused mostly on the development of novel formulations or combinations of drugs. However, HIV-1 and SRLVs infections are incurable with current therapies. Therefore, new drug approaches are needed^110^, which makes research on phytocompounds crucial for the development of effective therapeutics. Furthermore, the biodiversity of Brazilian flora offers a great opportunity for research on the use of plant compounds, including those with antiviral potential that may be used in human and veterinary medicine in alternative or complementary manners^111^.

In conclusion, the ethanolic fraction of ethyl acetate from both Meliaceae species showed better efficacy against CLV in colostrum and milk. Despite the lack of complete elimination of CLV, these natural products are interesting alternatives for the treatment of retroviral infections, and further studies are necessary for technical validation.

## Material and methods

### Bioethical aspects

**Figure 8:**
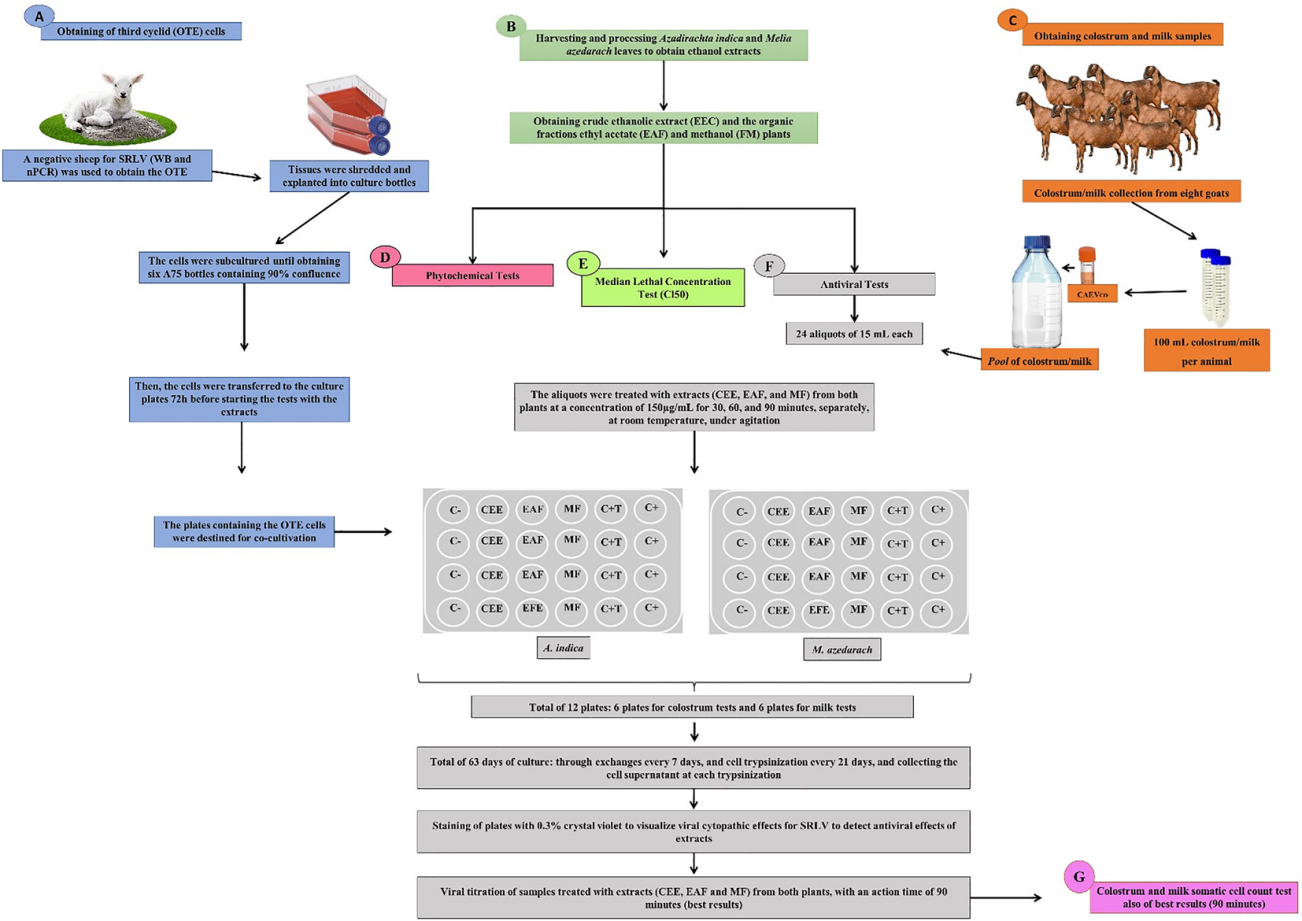
Illustration of the experimental design adopted in the evaluation antiviral in vitro activity ethanolic extracts of *Azadirachta indica* and *Melia azedarach* against the caprine lentivirus in colostrum and milk. OTE: ovine third eyelid; SRLV: Small Ruminant Lentiviruses; WB: Western Blot; *n*PCR: nested-Polymerase Chain Reaction; CAEV_CO_: standard viral strain; C-: Negative Control; C + T: Positive Control of Treatments; C+: Positive Control.

This project was submitted and approved by the Ethics Committee for the Use of Animals (CEUA) of Embrapa Goats and Sheep (protocol number 002/2018), following the guidelines of the National Council for Control of Animal Experimentation (CONCEA, law 11794 of October 8, 2008) and subsequent normative resolutions. The steps of this study are illustrated in Fig. 8.

### Collection and processing of *Azadirachta indica *and *Melia azedarach* ethanolic extracts

*A. indica* and *M. azedarach* leaves were collected from the Ceará and Piauí states, respectively, in a total volume of 5 kg each. These were then identified in exsiccates with vouchers 18,898 and 18,897, respectively, in Professor Francisco José Abreu de Matos’s herbarium, State University of Acaraú Valley (UVA), Sobral, Brazil.

Following identification, the leaves were separated from the branches and dried at ambient temperature. Then, 2.025 kg of *A. indica* and 1.26 kg of *M. azedarach* were submerged in 12 L and 13 L of 96% ethyl alcohol, respectively, and maintained for seven days in sealed recipients. Afterwards, the obtained solutions were subjected to a roto-evaporation process until 80–90% of the solvent was evaporated. The concentrate was maintained in a water bath to evaporate the remaining solvent until a pasty consistency was obtained, which formed the crude ethanolic leaf extracts of both the plants^57^.

Organic fractions of ethyl acetate and methanol were obtained via preparative vacuum-filtration chromatography. Thus, 25 g of each crude extract was added to 50 g of silica gel (1:2 ratio). Then, using a Buchner funnel, these mixtures were subjected to vacuum filtration and eluted with the organic solvents ethyl acetate and methanol, resulting in the respective fractions. The products were roto-evaporated and subjected to a water bath for complete evaporation of solvents, and the ethyl acetate and methanol fractions from both Meliaceae plants were obtained and stored at 4 °C until use.

### Mean lethal concentration toxicity test (LC_50_)

#### Cultivation of *Artemia salina* Leach cysts

*Artemia salina* Leach cysts were purchased from a specific store that sells products of animal origin. For cyst cultivation, a plastic beaker measuring approximately 2 L was used, containing 1000 mL of distilled, chlorine-free water, and 15 g of sea salt. Then, approximately 2.5 g of sodium bicarbonate was added until the pH stabilized at 8.0. Then, 5 g of *A. salina* cysts was immersed in this saline solution for hatching; the cysts were kept at a constant temperature of 25 °C, for 48 h, under white light and constant aeration to maintain the O_2_ levels in the medium^29^. After this period, the larvae hatched, reached the nauplii stage, and were subjected to test protocol for the average lethal concentration with crude extracts of *A. indica* and *M. Azedarach* and their respective fractions of ethyl acetate and methanol.

### Concentration toxicity test (LC_50_)

12-well plates were used for the tests. 2 mL of saline solution with 1% DMSO was added to each plate at concentrations of 100, 500, and 1000 µg/mL of extracts from both plants in the respective fractions. A total of 27 wells (approximately four plates) were used. For the test, treatments were ordered as indicated in Table 5, containing three replicates and n = 10 individuals per well^29^.

**Table 5:** Sampling distribution of *Artemia salina* Leach nauplii in the LC_50_ test with the crude extracts of *Azadirachta indica* and *Melia azedarach* and their respective fractions of ethyl acetate and methanol.

	100 µg/ml	500 µg/ml	1000 µg/ml	Control 1: saline solution	Control 2: DMSO1%	Control 3: NaClO 1%
*Azadirachta indica (neem)*						
CEE	Three repetitions and n = 10 in each well					
EAF						
MF						
*Melia azedarach (chinaberry tree)*						
CEE	Three repetitions and n = 10 in each well					
EAF						
MF						

DMSO, dimethyl sulfoxide; NaClO, sodium hypochlorite; CEE, crude ethanolic extract; EAF, ethyl acetate fraction; MF, methanol fraction.

After preparing the plates, they were incubated at 25 °C for 24 h under white light. After this period, the number of live and dead nauplii in each treatment group was counted. Subsequently, the data were analyzed to determine the toxicity and percentage of mortality of 50% of the larvae (50) using the probit analysis method using IBM SPSS 21 software.

### MTT (3-4,5-dimethyl-thiazol-2-yl-2,5-diphenyltetrazolium bromide) test for cell viability

The MTT assay was based on the methodology described by Dias et al.^43^ A cell suspension was prepared at a concentration of 2.0 × 10^5^ cells/mL in 12 96-well plates. The samples were then incubated in a CO_2_ oven for 24 h. Afterward, the wells were washed with 200 µl sterile PBS-1X at 37 °C. Then, the crude ethanolic extract (CEE), ethyl acetate fraction (EAF), and methanol fraction (MF) of *A. indica* and *M. azedarach* were added at concentrations of 10, 100 and 1000 µg/mL, and 6 repetitions, respectively. The plates were then kept in a CO_2_ oven for 24, 48, or 72 h. After each period, the wells were washed with 200 µl of buffer sodium phosphate (BSP) of each concentration, and 100 µl of the MTT solution (0.5 mg/mL) was added. They were then placed in a CO_2_ environment for four hours. Subsequently, the MTT solution was removed from the plates, and 100 µL of DMSO was added to each well, and each plate was shaken for 5 min. The absorbance at 570 nm was measured using a spectrophotometer.

Statistical analysis of absorbance was performed using mixed Repeated Measures Analysis of Variance, with the assumption of normality verified using the Shapiro–Wilk test and the assumption of sphericity of the variance–covariance matrix verified using the sphericity test of Mauchy. To verify the interaction between groups, time, and concentrations, we performed multiple comparisons of pairs of means with Bonferroni correction, as described by Marôco^112^, with a significance level of 5%. Statistical tests were performed using the IBM SPSS 21 software.

### Colostrum and milk samples

The experiment was performed using animals from the dairy flock of Embrapa Goats and Sheep in Sobral City, Ceará, Brazil. Approximately 100 mL of colostrum was collected from sterile recipients of eight goat nannies that recently gave birth and were positive for SRLV via nested polymerase chain reaction (nPCR)^113^. The first round of nPCR was performed, followed by a second round to amplify a final fragment of 185 base pairs (bp) of proviral DNA, which corresponds to the SRLV *gag* gene.

All oligonucleotide primers (*gag*1 CAAGCAGCAGGAGGGAGAAGCTG, *gag*2 TCCTACCCCCATAATTTGATCCAC, *gag*3 GTTCCAGCAACTGCAA ACAGTAGCAATG, and *gag*4 ACCTTTCTGCTTCTTCATTTAATTTCCC) were produced based on the standard CAEV-Co sequence (M33677.1)^114^.

In addition to the tested samples and for each round of amplification, a negative control (without DNA) and a positive control referring to CAEV_CO_.

The nPCR reactions were performed in a thermocycler (BIO-RAD, T100TM Thermal Cycler) in a total volume of 50 μL, containing buffer (10 mM tris–HCl, 50 mM KCl and 1.5 mM MgCl_2_—Sigma-Aldrich^®^, USA), 100 μM of each deoxynucleotide triphosphate (dNTP; Sigma-Aldrich^®^, USA), 20 pmol of each primer, 2U of Taq Platinum DNA polymerase (Thermo Fisher^®^, USA); 3 μL of sample in the first round and 1 μL of first round product in the second round. Amplification by nPCR was performed at 94 °C for five minutes, 35 cycles of 94 °C for one minute, 56 °C for one minute and 72 °C for 45 s; and a final extension at 72 °C for seven minutes. The amplified samples and controls (positive and negative) were subjected to electrophoresis in 2% agarose gel (Sigma-Aldrich^®^, USA), stained with ethidium bromide (Sigma-Aldrich^®^, USA), and visualized using an ultraviolet transilluminator (UVP, Benchtop UV Transiluminator M-26)^115^.

The samples were combined in a pool. This solution was reinfected with 450 µL of the standard CLV strain (CAEV_CO_, kindly provided by the Federal Rural University of Pernambuco (UFRPE) and derived from the *Laboratoire Associé de Recherches sur les Petits Ruminants*–INRA–ENVL, France) with an initial titer of 10^4.8^ TCID_50_/mL for 60 min at 37 °C under agitation. The same methodology was followed for the goat milk samples.

### Treatment of colostrum and milk samples

Crude ethanolic leaf extracts from *A. indica* and *M. azedarach* and ethyl acetate, and methanol fractions were diluted in dimethyl sulfoxide (DMSO) at 0.5%. Then, these solutions were added to colostrum/milk samples in the concentration of 150 µg/mL for 30, 60 and 90 min, individually. In addition, control treatments for colostrum and milk (no extract addition) were prepared. The samples were centrifugated at 3000*g* for 15 min at 4 °C. Somatic cells from colostrum/milk (SCC/SCM) were obtained with the methodology described by Karanikolaou et al*.*^116^ and submitted to co-culture with OTE cells.

### Co-culture with third eyelid (OTE) cells

For the co-culture, 12 24-well plates were prepared 72 h before the beginning of this step with the addition of OTE^41^ cells at a concentration of 2.5 × 10^5^ cells/mL. After confluence of approximately 80% OTE cells, SCC/SMC were distributed in plates at the same concentrations with four repetitions. In the wells, 500 µL of minimum essential medium (MEM) was added along with 2% amphotericin B, 3% penicillin and streptomycin, 1% gentamycin, and 5% fetal calf serum (FCS). Per plate, eight control wells were divided in a part with only OTE cells, negative control wells (C^−^), and the other half with positive controls (C^+^P) composed of OTE cells infected with the CLV standard strain. The plates were then incubated in a 5% CO_2_ environment at 37 °C. Media were replaced at seven-days intervals and cellular trypsinization was performed every 21 days. After 63 days of culture, the cell supernatant was collected and the wells were stained with crystal violet (0.1%) for visualization of viral cytopathic effects^19^.

### Titration of samples with antiviral activity

After visualization of viral cytopathic effects, samples with the best antiviral results in colostrum and milk were subjected to viral titrations^117^.

For this procedure, supernatants from the last collection of OTE + SCC/SCM co-culture cells were titrated in a microplate via decimal dilutions in minimum essential means (MEM) without fetal bovine serum (SFB) in four repetitions per dilution. For every 50 µL of viral dilution, 50 µL of OTE cell suspension at a concentration of 2.5 × 10^5^ cells/mL was added. Positive (OTE cells, MEM, and CLV with known titers) and negative (OTE cells and MEM) control wells were prepared. Microplates were incubated at 37 °C with 5% CO_2_ for 14 days with daily observations for typical cytopathic effects (CPE) and stained with 0.1% crystal violet. The titer was calculated according to the method described by Reed and Muench^117^. It was defined as the reciprocal of the highest dilution that was present 14 days after inoculation and syncytia in 50% of the inoculated wells^117^.

### Somatic cell count (SCC) of colostrum and milk samples

The colostrum and milk were in direct contact with the extracts of *A. indica* and *M. azedarach* for a maximum period of 90 min, after which the somatic cells contained in this material were obtained and used in the co-culture. Thus, for a detailed analysis, the somatic cell count (SCC) was performed according to the methodology recommended by Zeng et al.^118^.

To perform this test, approximately 100 mL of colostrum from eight goats (the same goats as in the initial experiment) was collected, and a pool of this material was formed, which was divided into 16 aliquots of 15 mL each. Colostrum samples were treated with 150 µg/mL crude extract from each plant and two respective fractions. Milk treatment was performed 30 days following the same methodology.

A clean and degreased histological slide was used for each colostrum and milk sample. Four smears of 1.0 cm^2^ were made on each slide, containing 0.01 mL of the samples. The slides were dried at room temperature and fixed using a Bunsen burner flame. Subsequently, the slides were immersed in xylene for three minutes, drained, and dried. The cells were then stained for six minutes with MGP (pyronine Y-methyl green; Sigma-Aldrich). For the reading, 50 fields of two smears from each slide were analyzed under an optical microscope in a 100× objective with the aid of immersion oil. The result was calculated by calculating the mean of the results obtained multiplied by the microscope's work factor, which is expressed as the number of somatic cells per mL of colostrum/milk (SC/mL), comparing the means of the treatments with the controls, using the Tukey test (ANOVA—BioEstat Software 5.3).

### Phytochemical testing of *Azadirachta indica *and *Melia azedarach* extracts by UPLC-HRMS (ultra-performance liquid chromatography coupled to high resolution mass spectrometry)

The extracts and their fractions were analyzed by UPLC-HRMS using a method previously described by Carvalho et al.^119^, with modifications. Prior to analysis, the samples were cleaned up through Supel-clean SPE-C18 cartridges (500 mg, Supelco, St. Louis, MO, USA) and posteriorly filtered with 0.22 μm PTFE syringe filters (Simplepure, Plano, TX, USA). Chromatographic separations were performed on an Acquity/Xevo UPLC-ESI-qTOF system (Waters Co., Milford, MA, USA), equipped with an Acquity UPLC BEH C18 column (Waters, 150.0 × 2.1 mm × 1.7 μm) at 40 °C. The mobile phase was composed of water and acetonitrile, both containing 0.1% formic acid, ranging from 2 to 95% acetonitrile for 22 min at a flow of 0.22 mL/min. The samples were filtered and injected into aliquots of 5.0 μL. The mass accuracy and reproducibility were maintained by infusing a 0.2 ng/µL leucine–enkephalin solution ([M−H]^−^ ion at m/z 556.2771) through LockSpray (Waters Corporation™) at a flow rate of 20 µL/min. MS data were recorded for m/z values in the range 110–1180 Da in both ionization modes. The compounds were tentatively characterized using the molecular formula provided by MassLynx 4.1 software on their accurate masses (error < 5 ppm), isotopic patterns (i-fit), and MS fragmentation patterns, as well as a literature survey of previous occurrences in the species studied and/or the Meliaceae family using the Scifinder Scholar database. Additionally, compounds were identified by comparison with reference standards when available.
